# An ANOCEF Genomic and Transcriptomic Microarray Study of the Response to Irinotecan and Bevacizumab in Recurrent Glioblastomas

**DOI:** 10.1155/2014/282815

**Published:** 2014-04-02

**Authors:** Julien Laffaire, Anna Luisa Di Stefano, Olivier Chinot, Ahmed Idbaih, Jaime Gallego Perez-Larraya, Yannick Marie, Nadia Vintonenko, Blandine Boisselier, Patrizia Farina, Jean-Yves Delattre, Dominique Figarella-Branger, Jérôme Honnorat, Marc Sanson, François Ducray

**Affiliations:** ^1^Programme Cartes d'Identité des Tumeurs (CIT), Ligue Nationale Contre le Cancer, Paris, France; ^2^AP-HP, Groupe Hospitalier Pitié-Salpêtrière, Service de Neurologie Mazarin, Paris, France; ^3^INSERM, U975, Centre de Recherche de l'Institut du Cerveau et de la Moelle, Paris, France; ^4^Faculté de Médecine Pitié-Salpêtrière, Université Pierre & Marie Curie Paris VI, CNRS UMR 7225 and UMR-S975, Paris, France; ^5^Neuro-Oncology Unit, C. Mondino National Neurological Institute, Pavia, Italy; ^6^Assistance Publique-Hôpitaux de Marseille, Service de Neuro-Oncologie, Centre Hospitalier Universitaire Timone, 264 rue Saint Pierre, 13385 Marseille Cedex 05, France; ^7^Aix-Marseille Université, Inserm, CRO2 UMR_S 911, 13385 Marseille, France; ^8^ANOCEF (Association des Neuro-Oncologues d'Expression Française (French Speaking NeuroOncologists' Association)), France; ^9^Medical Oncology 1, Venetian Oncology Institute-IRCCS, Padua, Italy; ^10^APHM, Hôpital de la Timone, Service d'Anatomie Pathologique et de Neuropathologie, 13385 Marseille, France; ^11^Service de Neuro-oncologie, Hôpital Neurologique, Hospices Civils de Lyon, 59 Boulevard Pinel, 69394 Lyon Cedex 3, Lyon, France; ^12^Université de Lyon, Claude Bernard Lyon 1, Lyon, France; ^13^Lyon Neuroscience Research Center, INSERM U1028/CNRS UMR 5292, Lyon, France

## Abstract

*Background*. We performed a retrospective study to assess whether the initial molecular characteristics of glioblastomas (GBMs) were associated with the response to the bevacizumab/irinotecan chemotherapy regimen given at recurrence. * Results*. Comparison of the genomic and gene expression profiles of the responders (*n* = 12) and nonresponders (*n* = 13) demonstrated only slight differences and could not identify any robust biomarkers associated with the response. In contrast, a significant association was observed between GBMs molecular subtypes and response rates. GBMs assigned to molecular subtype IGS-18 and to classical subtype had a lower response rate than those assigned to other subtypes. In an independent series of 33 patients, neither EGFR amplification nor CDKN2A deletion (which are frequent in IGS-18 and classical GBMs) was significantly associated with the response rate, suggesting that these two alterations are unlikely to explain the lower response rate of these GBMs molecular subtypes. * Conclusion*. Despite its limited sample size, the present study suggests that comparing the initial molecular profiles of responders and nonresponders might not be an effective strategy to identify biomarkers of the response to bevacizumab given at recurrence. Yet it suggests that the response rate might differ among GBMs molecular subtypes.

## 1. Background

In recurrent glioblastomas (GBMs), studies have shown a high response rate (30–50%) to bevacizumab, a human monoclonal antivascular endothelial growth factor (VEGF) antibody, administered alone or in combination with irinotecan, demonstrating a 35–50% estimated 6-month progression-free survival (PFS) [1–3]. Simple biomarkers that would help in selecting patients most likely to benefit from bevacizumab would be very helpful, but no such markers are available to date. In the present study, we hypothesized that the response to bevacizumab plus irinotecan given at recurrence might be related to the molecular characteristics of the initial tumor. To identify predictive biomarkers, we compared the initial GBM genomic and gene expression profiles of responders and nonresponders to bevacizumab plus irinotecan given at the time of recurrence.

## 2. Methods

### 2.1. Patients

We retrospectively identified responders and nonresponders to bevacizumab/irinotecan chemotherapy. This study was approved by the ANOCEF review board. All patients who underwent a genetic analysis of tumor samples collected for this study signed a written informed consent form. The patients' clinical characteristics are summarized in Table 1 and see additional Table  1 in Supplementary Material available online at http://dx.doi.org/10.1155/2014/282815. All of the 25 patients included in this study had* de novo* GBM according to the 2007 World Health Organization Classification [4] and were initially treated according to the Stupp regimen [5]. To exclude patients with possible pseudoprogression, only those patients with a progression occurring more than 3 months after the end of the radiochemotherapy treatment were selected [6]. Patients received bevacizumab (10 mg/kg) plus irinotecan (125 mg/m^2^) every two weeks either at the first (*n* = 15), second (*n* = 9), or third (*n* = 1) recurrence (chemotherapy details are available in additional Table  1). To identify clinically meaningful biomarkers of the response, the patients were considered to be responders if they achieved a complete or partial response according to RANO criteria [6] and presented more than 6-month progression-free survival (PFS); the patients were considered to be nonresponders if they progressed within 4 months.

### 2.2. Samples

The samples were provided as snap-frozen sections of the areas immediately adjacent to the region used for the histopathological diagnosis. Only samples representative of the tumor and from which high-quality DNA and/or RNA could be obtained were selected (*n* = 25). A total of 21 samples were available for the genomic Illumina SNP array study, which included samples from 8 responders and 13 nonresponders. The gene expression array study was performed on 23 samples (including 19 samples common to the SNP array study): 11 responders and 12 nonresponders.

### 2.3. Genomic and Gene Expression Data

#### 2.3.1. RNA and DNA Extraction

Total RNA was extracted using the RNeasy Lipid Tissue Mini Kit (Qiagen), and DNA was extracted using the QIAamp DNA Mini Kit (Qiagen) following the manufacturer's instructions. Both the RNA and DNA were assessed for integrity and quantity, following stringent quality control criteria (CIT program protocols http://cit.ligue-cancer.net/). The genomic and gene expression analyses were performed using R software (http://www.R-project.org/).

#### 2.3.2. Gene Expression Arrays

The gene expression arrays were performed using the IGBMC microarray platform (Strasbourg, France). Total RNA was amplified, labeled, and hybridized to the Affymetrix Human Genome U133 plus2 GeneChip, following the manufacturer's protocol (Affymetrix, Santa Clara, CA, USA). The microarrays were scanned using an Affymetrix GeneChip Scanner 3000, and the raw intensities were quantified from the subsequent images using GCOS 1.4 software (Affymetrix). The data were normalized using the robust multiarray average method implemented in the R package affinity [9].

Unsupervised hierarchical clustering analysis was performed using the Pearson correlation metric. Only probesets with an Affymetrix annotation class A and located on autosomes were considered. Differences between the sample clusters were tested using the Chi-squared test, and genes differentially expressed between the tumors of responder and nonresponder patients were assessed using the *t*-test followed by Benjamini and Hochberg correction. The analyses of the gene sets using KEGG and Biocarta pathways and Gene Ontology terms, Molecular Signature Database gene sets, and Stanford Microarray Database gene sets were performed on the 1000 most differentially expressed genes (500 genes upregulated in responders and 500 genes upregulated in nonresponders) using hypergeometric tests [10]. We used the published centroid-based classifier of Verhaak et al. to classify our samples according to their system [7]. Samples were assigned to one of the six molecular subtypes of gliomas (called intrinsic glioma subtypes (IGS)) described by Gravendeel et al. [8] using ClusterRepro (an R package; http://crantastic.org/packages/clusterRepro) [11].

#### 2.3.3. Genomic Arrays

The genomic arrays were performed using the IntegraGen Platform (Evry, France). DNA was hybridized to Illumina SNP Human CNV370 chips according to the instructions provided by the array manufacturer (Illumina, San Diego, CA). The raw fluorescent signals were imported into Illumina BeadStudio software and normalized as previously described [12] to obtain the log R ratio (LRR) and B Allele Frequency (BAF) for each SNP. A supplemental normalization procedure tQN [13] was applied to correct for dye bias. The genomic profiles were then segmented using the circular binary segmentation algorithm (DNAcopy package, Bioconductor) [14] into the LRR and BAF data separately, as previously described [13, 15]. The absolute copy number and genotype status of the segments were then determined using the genome alteration print (GAP) method [15].

The data are available in the ArrayExpress database (http://www.ebi.ac.uk/arrayexpress/), ArrayExpress accession: E-MTAB-951.

#### 2.3.4. RT-PCR

The gene expression of NPTX2, EPHA7, SOCS2, PDGFD, PRKCZ, and ENPP4 in the tumors and nontumor control tissue were analyzed using UPL probe real-time quantitative polymerase chain reaction (QPCR) analysis. The reference gene was PPIA. The sequences of the primers and probes are listed in additional Table  2. The real-time QPCR reactions were performed as follows: 1X LightCycler 480 Probes Master (Roche Applied Science), 4 pmoles each primer, 2 pmoles Universal ProbeLibrary Set, Human, and 8 ng cDNA. The real-time QPCR cycles were as follows: initial denaturation at 95°C for 110 minutes and 45 cycles of 95°C for 10 seconds and annealing at 60°C for 30 minutes. The 2-DeltaDeltaCT method was used to determine the relative expression levels. The calculation of the relative amounts of the studied transcript compared to the reference transcript was performed using the LightCycler 480 Software (Roche applied science). The final results were expressed as a ratio of the expression levels of the studied gene and reference in the sample, normalized to the ratio of the reference gene expression in the calibration RNA.

### 2.4. Independent Data Set

An independent series of 33 GBMs from the Salpêtrière database treated with the bevacizumab/irinotecan combination at recurrence (31 out of 33 were treated at first recurrence) was used to assess the impact of the* CDKN2A* homozygous deletion and* EGFR* amplification. These alterations were assessed in the initial tumor using CGH arrays as previously described [16]. The response according to RANO criteria was assessable in 29 of the patients. RNA was available for 7 of the responders and 11 nonresponders and was used to study NPTX2, EPHA7, SOCS2, PDGFD, PRKCZ, and ENPP4 gene expression using RT-PCR.

## 3. Results

### 3.1. Patients' Characteristics

Twelve responders and thirteen nonresponders were included. All of the MRIs were reviewed. All of the patients exhibited an evaluable disease at the initiation of bevacizumab plus irinotecan treatment. The patients' characteristics are shown in Table 1. After bevacizumab/irinotecan onset, the responders had a longer progression-free survival (PFS) and overall survival (OS) than the nonresponders. The OS since diagnosis was also significantly longer for the responders (Table 1).

### 3.2. Responders and Nonresponders Have Very Similar Genomic and Gene Expression Profiles

**Figure 1:**
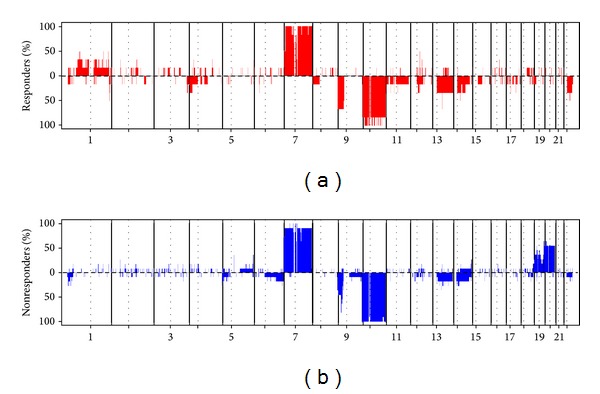
Genomic profiles of responders and nonresponders. Genomic profiles of responders and nonresponders to the bevacizumab/irinotecan regimen. For each chromosome, the telomere of the short arm is on the left and the telomere of the long arm is on the right. The *y*-axis corresponds to the frequency of gains and losses in each group of patients.

The comparison of the genomic profiles (gains, losses, homozygous deletions, and amplifications) of the responders (*n* = 8) versus nonresponders (*n* = 13) demonstrated only slight genomic differences (Figure 1, additional Tables  3a and  3b), with the most consistent being an entire chromosome 20 gain that was significantly more frequent in the nonresponders (Fisher's exact test *P* = 0.04).* EGFR* amplification (9/13 in nonresponders versus 4/8 in responders) and* CDKN2A* locus homozygous deletion (8/13 in nonresponders versus 4/8 in responders) were also more frequently observed in nonresponders, but the difference was not significant.

Similarly, the comparison of the gene expression profiles of the responders (*n* = 11) and nonresponders (*n* = 12) demonstrated only few differences. Sixty probe sets (fifty-one in responders and nine in nonresponders) were differentially expressed, with a *t*-test *P* value <0.05 and a fold change above 2, though with a very high (95%) false discovery rate (additional Table  4). Neither the expression of VEGF nor its receptors were associated with the response to the treatment. Using RT-PCR we studied the expression of 6 genes implicated in angiogenesis and overexpressed in responders (ENPP4, PRKCZ, and EPHA7) or nonresponders (NPTX2, SOCS2, and PDGFD) in an independent series of 7 responders and 11 nonresponders. EPHA7 [17] is implicated in endothelial tubulogenesis, and PRKCZ has been implicated in VEGF transcriptional activation [18]. NPTX2 has been shown to be overexpressed in edematous versus nonedematous gliomas in the absence of increased VEGF expression [19]. PDGFD is a proangiogenic factor [20], and SOCS2 is involved in IGF1R signaling and is also a proangiogenic factor [21]. However, with the exception of SOCS2, we failed to confirm similar overexpression in the responders/nonresponders that was significant in this independent series (additional Table  5). Lastly, the pathway analysis performed on the 1000 genes that were most differentially expressed (500 genes upregulated in responders and 500 genes upregulated in nonresponders) demonstrated that these gene lists were significantly enriched in genes with different ontologies (additional Tables  6 and  7). The list of upregulated genes in the responders was significantly enriched in genes upregulated in the normal brain, whereas the list of upregulated genes in the nonresponders was enriched in genes that have been shown to be upregulated during hypoxia [22] and also in genes that might be targets of the transcription factor HIF1.

### 3.3. GBMs Molecular Subtypes Are Associated with Different Response Rates

**Figure 2:**
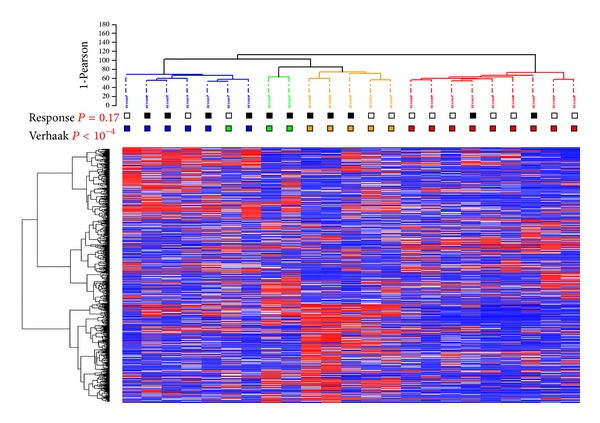
Unsupervised hierarchical clustering of the 23 GBMs. The heatmap was constructed using the 2365 probesets (quantile 0.95), with the greatest robust coefficient of variation between the tumor samples. The samples and genes were clustered using Ward's linkage and Pearson's correlation coefficient. For each probe set, the lowest and highest intensity values are displayed in blue and red, respectively. Response: black = responder, white = nonresponder. Verhaak = class according to Verhaak et al.'s classification [7]: neural = green, classical = red, mesenchymal = blue, and proneural = orange.

**Figure 3:**
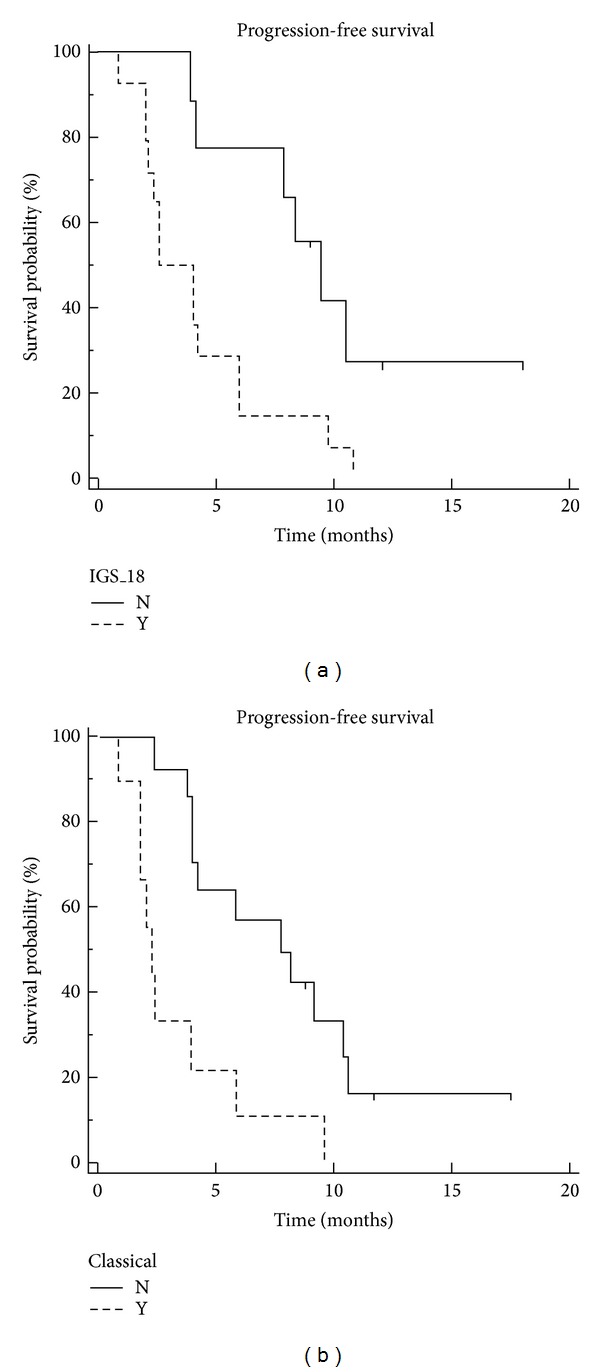
Progression-free survival according to Gravendeel et al. [8] and Verhaak et al. [7] molecular subtypes. GBMs assigned to IGS-18 (dashed line) had a shorter PFS after bevacizumab/irinotecan than those assigned to IGS-22 and IGS-23 (plain line) (3.2 months versus 9.4 months, *P* = 0.01). GBMs classified as classical (dashed line) had a shorter PFS than those classified as nonclassical (2.2 months versus 8.3 months, *P* = 0.003).

As responders and nonresponders had very similar gene expression profiles, we hypothesized that there might be several subgroups of responders and nonresponders. To test this hypothesis, we performed an unsupervised hierarchical clustering analysis of the 23 GBMs included in the gene expression study. As shown in Figure 2, three main subgroups were identified. This clustering was robust and conserved across different gene lists and clustering methods. However, none of the three clusters was enriched in responders or nonresponders, and some responders and nonresponders could have very similar gene expression profiles. Therefore, to assess whether transcriptomic subgroups of GBMs previously identified in larger series of patients were associated with a specific pattern of response to the bevacizumab/irinotecan regimen, we classified our 23 samples according to the transcriptomic classifications of Gravendeel et al. [8] and of Verhaak et al. [7] and estimated the response rate in each subgroup. According to Gravendeel et al. [8], 14 GBMs were assigned to molecular subtype 18 (IGS-18), 3 to molecular subtype 22 (IGS-22), and 6 to molecular subtype 23 (IGS-23). According to Verhaak et al. [7], 9 GBMs were classified as classical, 6 as mesenchymal, 5 as proneural, and 3 as neural. The 9 classical GBMs were also assigned to IGS-18 which in addition consisted of 3 neural and 2 proneural GBMs. Interestingly, the GBMs assigned to IGS-18 were more frequently not responsive than the GBMs assigned to IGS-22 or IGS-23 (10/14 versus 2/9, Fisher's exact test *P* value = 0.03) and a similar trend was observed for classical versus nonclassical GBMs (7/9 versus 5/14, Fisher's exact test *P* value = 0.09). Conversely, IGS-18 GBMs had a shorter PFS after bevacizumab/irinotecan than IGS-22/23 GBMs (3.2 months versus 9.4 months, *P* = 0.01) and classical GBMs had a shorter PFS than nonclassical GBMs (2.2 months versus 8.3 months, *P* = 0.003) (Figure 3). Overall survival after bevacizumab/irinotecan also tended to be shorter in IGS-18 than in IGS-22/23 GBMs and in classical than nonclassical GBMs (7 months versus 18.9 months, *P* = 0.06 and 6.6 months versus 14.3 months, *P* = 0.06).

### 3.4. Neither EGFR Amplification Status Nor CDKN2A Locus Homozygous Deletion Status Is Associated with the Response Rate, the Progression-Free Survival, or the Overall Survival after Bevacizumab/Irinotecan Initiation

Because, in our series,* EGFR* amplification and* CDKN2A* homozygous deletion were more frequent in IGS-18 GBMs than in IGS-22/23 GBMs (11/14 versus 0/5, Fisher's exact test *P* value <0.01 and 10/14 versus 1/5 Fisher's exact test *P* value = 0.1, resp.) and also more frequent in classical than in nonclassical GBMs (9/9 versus 2/10, Fisher's exact test *P* value < 0.01 and 10/14 versus 1/5 Fisher's exact test *P* value = 0.02, resp.), we decided to evaluate the impact of these two genomic abnormalities in an independent series, in order to assess if these genomic abnormalities contribute to the lower response rate of IGS-18 and classical GBMs. This independent series comprised 33 GBMs from the Salpêtrière database treated with the combination of bevacizumab/irinotecan at recurrence and for whom the* CDKN2A* locus homozygous deletion and* EGFR* amplification status were available in the initial tumor. The patients' characteristics are shown in Table 2. However, we did not observe any significant association between* EGFR* amplification and/or* CDKN2A* deletion status and the response rate to bevacizumab/irinotecan, the PFS, or the OS after bevacizumab/irinotecan initiation.

## 4. Discussion

Several studies have identified radiological, plasmatic, or clinical markers of the response to bevacizumab [23–25]. The objective of the present study was to identify biomarkers predictive of the response to bevacizumab/irinotecan given at GBM recurrence based on the transcriptomic and genomic characterization of the initial tumor. Given the dramatically different clinical and radiological response patterns to this treatment, we hypothesized that the comparison of a limited series of well-selected responders and nonresponders would be sufficient to identify robust and clinically useful biomarkers if such markers do exist. However, although the responders and nonresponders had dramatically different response patterns, we found that the two groups of patients had very similar genomic and gene expression profiles and we failed to identify any robust predictive biomarker. There are several possible hypotheses to explain this finding. First, the genomic and transcriptomic characteristics of the initial tumor might not be predictive of the response to bevacizumab/irinotecan given at recurrence because the molecular profile of recurrent GBMs might have significantly changed. However, Sathornsumetee et al. demonstrated that the expression of VEGF and CA9 (a marker of hypoxia) assessed by immunohistochemistry in the initial GBM was associated with the response and survival, respectively, in patients receiving bevacizumab and irinotecan at recurrence [26]. Interestingly, we similarly found that the profile of nonresponders was enriched in genes upregulated during hypoxia, though not influenced by VEGF expression. A second hypothesis to explain the absence of major difference between the profiles of responders and nonresponders is that the criteria used for defining the responders and nonresponders in the present study were not appropriate. These criteria were chosen to discover biomarkers that might be clinically meaningful and that might identify responders that achieve both a radiological response and prolonged PFS (>6 months) and to differentiate these patients from those who progress rapidly, regardless of the radiological response. Another hypothesis (and we suggest the most likely) is that the comparison of responders and nonresponders (regardless of the criteria) might not be the best strategy to identify biomarkers of the response. Indeed, this strategy assumes that all of the responders and nonresponders share common characteristics, which might be inappropriate if there are not one but several subgroups of responders/nonresponders with different mechanisms of response or resistance. In fact, both Verhaak et al. and Gravendeel et al. demonstrated that this is likely to be the case, as they identified transcriptomic subgroups of GBMs that seem to display different patterns of response according to the treatment used [7, 8]. Furthermore, we previously found that mesenchymal GBMs were more likely to respond to radiotherapy, whereas classical GBMs were more likely to respond to first-line alkylating chemotherapy [10]. In our series, though it was not designed to study this association, we observed an interesting association between GBMs molecular classes and the response rates. Using Gravendeel et al. classification, GBMs assigned to IGS-18 had a lower response rate to bevacizumab/irinotecan than the GBMs assigned to IGS-22 and IGS-23 [8]. Using Verhaak et al. classification a similar trend was observed for classical GBMs [7] when compared to nonclassical GBMs. This is in agreement with the fact that IGS-18 GBMs are generally assigned to the classical subtype (9 out of 14 cases in our series) [27]. As* EGFR* amplification and* CDKN2A* deletion status are two genomic hallmarks of IGS-18 and classical GBMs, we next studied the impact of these two genomic abnormalities in an independent series of 33 patients. However, we did not identify any significant association with the response rate to bevacizumab/irinotecan suggesting that* EGFR* amplification and* CDKN2A* deletion are not responsible for the lower response rate of IGS-18 and classical GBMs to bevacizumab/irinotecan.

Taken together, our findings suggest that comparing the initial genomic and gene expression profiles of responders and nonresponders might not be an effective strategy to identify robust biomarkers of the response to bevacizumab/irinotecan given at recurrence. Yet, they also suggest that GBMs molecular subclasses are associated with the response to this treatment. This result however needs to be validated in a prospective and larger series of patients.

## Supplementary Material

Additional Table 1: Patients' clinical characteristics and classification according to Gravendeel and Verhaak classification. Additional Table 2: Primers used for RT-PCR. Additional Table 3: a) Main Genomic alterations significantly associated with the response to bevacizumab/irinotecan b) Complete list of genomic differences with a *P*-value < 0.05 in responders and non-responders. Additional Table 4: Complete list of genes differentially expressed with a *P*-value < 0.05 between responders and non-responders. Additional Table 5: Gene expression fold change in the initial and independent series of the 6 genes studied using RT-PCR. Additional Table 6: List of gene sets with a hypergeometric *P*-value < 0.05 in non-responders. Additional Table 7: List of gene sets with a hypergeometric *P*-value < 0.05 in responders.

## Figures and Tables

**Table 1:** Patient characteristics.

	Nonresponders	Responders	
Number of patients	13	12	
Median age (years) at diagnosis (range)	56 (37–69)	62 (57–72)	*t*-test *P* = 0.01
Biopsy/resection (%)	0/100	25/75	
Initial treatment (%)	RTCT (100%)	RTCT (100%)	
Median delay (months) between diagnosis and bev./iri. onset (range)	11 (7–22)	13 (5–27)	Ns
Recurrence number at bev./iri. onset			
First	8	7	
Second	4	5	
Third	1	0	
Median KPS at bev./iri. onset	70	80	Ns
Median PFS after bev./iri. onset (months)	2.4	9.4	*P* < 0.0001
Median OS after bev./iri. onset (months)	6.4	18.9	*P* = 0.0001
Median OS since diagnosis (months)	18.3	36.4	*P* = 0.002

RTCT: temozolomide radiochemotherapy; bev./iri.: bevacizumab/irinotecan chemotherapy; KPS: Karnofsky performance status; PFS: progression-free survival; OS: overall survival; ns: not significant.

**Table 2:** Characteristics of the 33 patients from the Salpêtrière database for whom the impact of EGFR amplification and CDKN2A locus homozygous deletion was assessed.

Characteristics of the 33 patients of the independent dataset	
Number of patients	33
Median age (years) at diagnosis (range)	59 (25–81)
Initial treatment (%)	RTCT (100%)
Median delay (months) between diagnosis and bev./iri. onset (range)	15 (3.5–60)
Recurrence number at bev./iri. onset	
First	31
Second/third	1/1
Response according to RANO	
Complete	4
Partial	11
Stable	7
Progression	7
Not assessable	3
EGFR amplification	13
CDKN2A homozygous deletion	12
Median PFS after bev./iri. onset (months)	5.5
Median OS after bev./iri. onset (months)	9.7
Median OS since diagnosis (months)	29

RTCT: temozolomide radiochemotherapy; bev./iri.: bevacizumab/irinotecan chemotherapy; KPS: Karnofsky performance status; PFS: progression-free survival; OS: overall survival; ns, nonsignificant.
